# 3-(2-Amino­ethyl)-2-anilinoquinazolin-4(3*H*)-one methanol hemisolvate

**DOI:** 10.1107/S1600536809045516

**Published:** 2009-11-04

**Authors:** Tao Gao, Yuan-Hong Jiao, Seik Weng Ng

**Affiliations:** a Faculty of Chemistry and Life Science, Xianning University, Xianning 437100, People’s Republic of China; b School of Chemistry and Material Engineering, Huangshi Institute of Technology, Huangshi 435003, People’s Republic of China; c Department of Chemistry, University of Malaya, 50603 Kuala Lumpur, Malaysia

## Abstract

The title methanol hemisolvated quinazolin-(3*H*)-one, C_16_H_16_N_4_O·0.5CH_3_OH, has an anilino substituent in the 2-position and an amino­ethyl substituent in the 3-position of the planar fused-ring system (r.m.s. deviation = 0.019 Å). The anilino N atom donates an intramolecular hydrogen bond to the amino­ethyl N atom. The mol­ecule and the solvent methanol mol­ecule are linked by N—H⋯N, N—H⋯O and O—H⋯O hydrogen bonds. The methanol mol­ecule is disordered over two equally occupied positions about a twofold rotation axis.

## Related literature

For the synthesis of this class of compounds, see: Yang *et al.* (2008 ▶). For the crystal structure of a chlorine-substituted derivative, see: Yang *et al.* (2009 ▶).
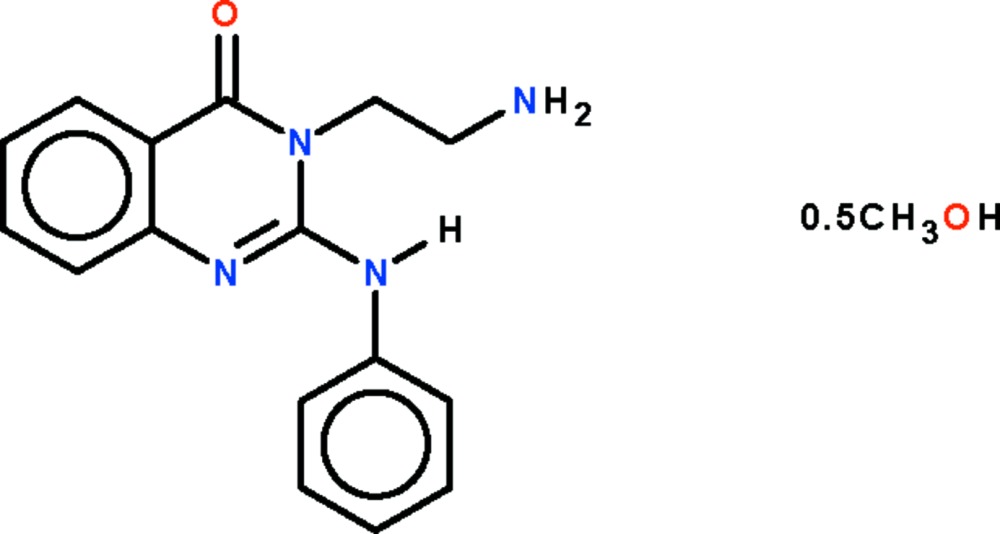


## Experimental

### 

#### Crystal data

C_16_H_16_N_4_O·0.5CH_4_O
*M* *_r_* = 296.35
Monoclinic, 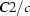

*a* = 19.5972 (11) Å
*b* = 12.2035 (7) Å
*c* = 12.8681 (8) Å
β = 103.301 (1)°
*V* = 2994.9 (3) Å^3^
*Z* = 8
Mo *K*α radiation
μ = 0.09 mm^−1^
*T* = 295 K
0.30 × 0.20 × 0.10 mm

#### Data collection

Bruker APEXII diffractometer
Absorption correction: none
14007 measured reflections
3399 independent reflections
2377 reflections with *I* > 2σ(*I*)
*R*_int_ = 0.073

#### Refinement

*R*[*F* ^2^ > 2σ(*F* ^2^)] = 0.062
*wR*(*F* ^2^) = 0.186
*S* = 1.09
3399 reflections
210 parameters
13 restraints
H-atom parameters constrained
Δρ_max_ = 0.55 e Å^−3^
Δρ_min_ = −0.29 e Å^−3^

### 

Data collection: *APEX2* (Bruker, 2007 ▶); cell refinement: *SAINT* (Bruker, 2007 ▶); data reduction: *SAINT*; program(s) used to solve structure: *SHELXS97* (Sheldrick, 2008 ▶); program(s) used to refine structure: *SHELXL97* (Sheldrick, 2008 ▶); molecular graphics: *X-SEED* (Barbour, 2001 ▶); software used to prepare material for publication: *publCIF* (Westrip, 2009 ▶).

## Supplementary Material

Crystal structure: contains datablocks global, I. DOI: 10.1107/S1600536809045516/bt5121sup1.cif

Structure factors: contains datablocks I. DOI: 10.1107/S1600536809045516/bt5121Isup2.hkl

Additional supplementary materials:  crystallographic information; 3D view; checkCIF report

## Figures and Tables

**Table 1:** Hydrogen-bond geometry (Å, °)

*D*—H⋯*A*	*D*—H	H⋯*A*	*D*⋯*A*	*D*—H⋯*A*
N3—H31⋯N4	0.88	2.04	2.811 (3)	146
N4—H41⋯O2^i^	0.88	2.13	2.990 (6)	168
O2—H2*O*⋯O1	0.84	2.01	2.755 (6)	147

Symmetry code: (i) 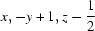.

**Fig. 1.**
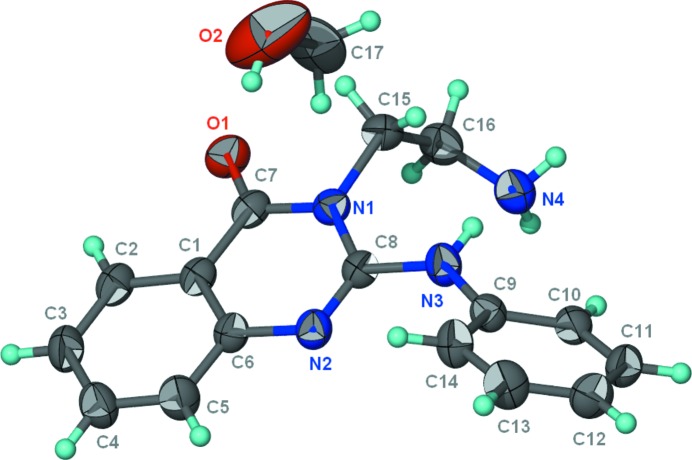
Anisotropic displacement ellipsoid plot (Barbour, 2001) of the title compound at the 70% probability level; hydrogen atoms are drawn as spheres of arbitrary radius.

## supplementary crystallographic information

### Experimental

To a THF (10 ml) solution of 2-ethoxycarbonyliminophosphorane (1.27 g, 3.0 mmol) was added phenylisocyanate (0.36 g, 3.0 mmol). The solution was set aside undisturbed for 6 h at 273 K. To this solution was added ethanolamine (0.18 g, 3 mmol) in THF (5 ml). The mixture was stirred overnight. The solvent was removed and the solid recrystallized from a chloroform/methanol (1/1) mixture to give colorless crystals in 80% yield; m.p. 433–434 K.

### Refinement

Carbon-bound H-atoms were placed in calculated positions (C—H 0.95 to 0.97 Å) and were included in the refinement in the riding model approximation, with *U*(H) set to 1.2 to 1.5*U*(C). The amino and hydroxy H atoms were similarly generated.

The methanol molecule is disordered over two equally occupied positions about a two-fold rotation axis. The C–O distance was restrained to 1.500±0.002 Å. The anisotropic displacemnt parameters of the methanolic O and C atoms were restrained to be nearly isotropic.

### Crystal data

**Table:**

C_16_H_16_N_4_O·0.5CH_4_O	*F*(000) = 1256
*M**_r_* = 296.35	*D*_x_ = 1.314 Mg m^−^^3^
Monoclinic, *C*2/*c*	Mo *K*α radiation, λ = 0.71073 Å
Hall symbol: -C 2yc	Cell parameters from 3816 reflections
*a* = 19.5972 (11) Å	θ = 2.4–25.9°
*b* = 12.2035 (7) Å	µ = 0.09 mm^−^^1^
*c* = 12.8681 (8) Å	*T* = 295 K
β = 103.301 (1)°	Block, colorless
*V* = 2994.9 (3) Å^3^	0.30 × 0.20 × 0.10 mm
*Z* = 8	

### Data collection

**Table:**

Bruker APEXII diffractometer	2377 reflections with *I* > 2σ(*I*)
Radiation source: fine-focus sealed tube	*R*_int_ = 0.073
graphite	θ_max_ = 27.5°, θ_min_ = 2.0°
ω scans	*h* = −17→25
14007 measured reflections	*k* = −15→14
3399 independent reflections	*l* = −16→16

### Refinement

**Table:**

Refinement on *F*^2^	Primary atom site location: structure-invariant direct methods
Least-squares matrix: full	Secondary atom site location: difference Fourier map
*R*[*F*^2^ > 2σ(*F*^2^)] = 0.062	Hydrogen site location: inferred from neighbouring sites
*wR*(*F*^2^) = 0.186	H-atom parameters constrained
*S* = 1.09	*w* = 1/[σ^2^(*F*_o_^2^) + (0.1044*P*)^2^ + 0.1504*P*] where *P* = (*F*_o_^2^ + 2*F*_c_^2^)/3
3399 reflections	(Δ/σ)_max_ = 0.001
210 parameters	Δρ_max_ = 0.55 e Å^−^^3^
13 restraints	Δρ_min_ = −0.29 e Å^−^^3^

### Fractional atomic coordinates and isotropic or equivalent isotropic displacement parameters (Å^2^)

**Table:**

	*x*	*y*	*z*	*U*_iso_*/*U*_eq_	Occ. (<1)
O1	0.68958 (7)	0.41303 (12)	0.81309 (11)	0.0573 (4)	
N1	0.73674 (8)	0.41620 (12)	0.66714 (11)	0.0424 (4)	
N2	0.84958 (8)	0.34284 (13)	0.67038 (11)	0.0458 (4)	
N3	0.77752 (9)	0.40366 (13)	0.51241 (12)	0.0498 (4)	
H31	0.7345	0.4239	0.4817	0.060*	
N4	0.63136 (9)	0.37965 (16)	0.44490 (14)	0.0632 (5)	
H41	0.6131	0.4263	0.3939	0.076*	
H42	0.6131	0.3143	0.4284	0.076*	
C1	0.80430 (10)	0.34133 (14)	0.83148 (13)	0.0414 (4)	
C2	0.81360 (11)	0.31401 (16)	0.93932 (14)	0.0503 (5)	
H2	0.7782	0.3290	0.9746	0.060*	
C3	0.87449 (12)	0.26533 (18)	0.99341 (15)	0.0576 (6)	
H3	0.8804	0.2465	1.0650	0.069*	
C4	0.92707 (11)	0.24457 (19)	0.94030 (15)	0.0589 (6)	
H4	0.9684	0.2115	0.9767	0.071*	
C5	0.91908 (11)	0.27202 (19)	0.83510 (16)	0.0573 (6)	
H5	0.9552	0.2579	0.8011	0.069*	
C6	0.85720 (10)	0.32115 (15)	0.77791 (13)	0.0427 (5)	
C7	0.73916 (10)	0.39143 (15)	0.77321 (14)	0.0437 (5)	
C8	0.79099 (10)	0.38642 (15)	0.61962 (14)	0.0414 (4)	
C9	0.82431 (10)	0.39287 (14)	0.44502 (14)	0.0426 (5)	
C10	0.79487 (10)	0.36669 (15)	0.33875 (14)	0.0462 (5)	
H10	0.7472	0.3515	0.3175	0.055*	
C11	0.83579 (12)	0.36311 (17)	0.26507 (15)	0.0530 (5)	
H11	0.8154	0.3462	0.1942	0.064*	
C12	0.90627 (13)	0.38417 (19)	0.29508 (18)	0.0614 (6)	
H12	0.9338	0.3820	0.2451	0.074*	
C13	0.93592 (12)	0.40868 (19)	0.40098 (18)	0.0616 (6)	
H13	0.9838	0.4225	0.4220	0.074*	
C14	0.89551 (11)	0.41293 (17)	0.47595 (16)	0.0534 (5)	
H14	0.9161	0.4292	0.5468	0.064*	
C15	0.67613 (10)	0.48235 (17)	0.61029 (15)	0.0516 (5)	
H15A	0.6922	0.5335	0.5634	0.062*	
H15B	0.6587	0.5248	0.6624	0.062*	
C16	0.61626 (11)	0.41579 (18)	0.54480 (17)	0.0586 (6)	
H16A	0.6080	0.3524	0.5857	0.070*	
H16B	0.5739	0.4599	0.5298	0.070*	
O2	0.5495 (3)	0.4637 (6)	0.7850 (7)	0.176 (3)	0.50
H2O	0.5856	0.4244	0.8006	0.211*	0.50
C17	0.4883 (4)	0.3949 (6)	0.7344 (11)	0.101 (3)	0.50
H17A	0.4553	0.3937	0.7792	0.152*	0.50
H17B	0.5037	0.3216	0.7254	0.152*	0.50
H17C	0.4662	0.4250	0.6660	0.152*	0.50

### Atomic displacement parameters (Å^2^)

**Table:**

	*U*^11^	*U*^22^	*U*^33^	*U*^12^	*U*^13^	*U*^23^
O1	0.0468 (8)	0.0701 (10)	0.0607 (8)	0.0019 (7)	0.0243 (7)	−0.0043 (6)
N1	0.0402 (9)	0.0437 (9)	0.0438 (8)	0.0002 (7)	0.0110 (7)	−0.0028 (6)
N2	0.0417 (9)	0.0570 (10)	0.0402 (8)	0.0011 (7)	0.0130 (7)	−0.0002 (6)
N3	0.0430 (9)	0.0665 (11)	0.0405 (8)	0.0019 (8)	0.0107 (7)	0.0053 (7)
N4	0.0614 (12)	0.0661 (12)	0.0558 (10)	0.0053 (9)	0.0006 (9)	−0.0015 (8)
C1	0.0435 (10)	0.0412 (10)	0.0411 (9)	−0.0070 (8)	0.0128 (8)	−0.0050 (7)
C2	0.0578 (13)	0.0540 (12)	0.0437 (10)	−0.0063 (10)	0.0214 (9)	−0.0038 (8)
C3	0.0697 (14)	0.0649 (13)	0.0384 (9)	−0.0019 (11)	0.0128 (10)	0.0028 (9)
C4	0.0490 (12)	0.0744 (15)	0.0494 (11)	0.0035 (11)	0.0035 (10)	0.0062 (9)
C5	0.0446 (12)	0.0782 (15)	0.0510 (11)	0.0058 (10)	0.0152 (9)	0.0035 (10)
C6	0.0424 (10)	0.0490 (11)	0.0378 (9)	−0.0038 (8)	0.0112 (8)	−0.0014 (7)
C7	0.0443 (11)	0.0438 (10)	0.0459 (10)	−0.0063 (8)	0.0164 (8)	−0.0070 (7)
C8	0.0413 (10)	0.0424 (10)	0.0415 (9)	−0.0040 (8)	0.0112 (8)	−0.0027 (7)
C9	0.0445 (11)	0.0432 (10)	0.0411 (9)	−0.0007 (8)	0.0122 (8)	0.0067 (7)
C10	0.0482 (11)	0.0448 (11)	0.0440 (9)	−0.0001 (8)	0.0072 (8)	0.0011 (7)
C11	0.0636 (14)	0.0570 (12)	0.0404 (9)	0.0007 (10)	0.0159 (9)	−0.0006 (8)
C12	0.0654 (15)	0.0711 (15)	0.0554 (12)	0.0043 (11)	0.0293 (11)	0.0041 (10)
C13	0.0466 (12)	0.0792 (16)	0.0620 (13)	−0.0042 (10)	0.0187 (10)	0.0085 (10)
C14	0.0490 (12)	0.0683 (14)	0.0423 (10)	−0.0097 (10)	0.0091 (9)	0.0032 (9)
C15	0.0507 (12)	0.0492 (12)	0.0548 (11)	0.0090 (9)	0.0119 (9)	−0.0007 (8)
C16	0.0445 (12)	0.0671 (14)	0.0618 (13)	0.0074 (10)	0.0071 (10)	0.0025 (10)
O2	0.109 (4)	0.189 (6)	0.231 (6)	−0.017 (4)	0.043 (4)	−0.115 (5)
C17	0.044 (6)	0.143 (5)	0.117 (8)	0.021 (4)	0.017 (5)	0.021 (5)

### Geometric parameters (Å, °)

**Table:**

O1—C7	1.227 (2)	C5—H5	0.9300
N1—C7	1.388 (2)	C9—C14	1.382 (3)
N1—C8	1.391 (2)	C9—C10	1.394 (2)
N1—C15	1.482 (2)	C10—C11	1.376 (3)
N2—C8	1.297 (2)	C10—H10	0.9300
N2—C6	1.383 (2)	C11—C12	1.370 (3)
N3—C8	1.360 (2)	C11—H11	0.9300
N3—C9	1.406 (2)	C12—C13	1.386 (3)
N3—H31	0.8800	C12—H12	0.9300
N4—C16	1.453 (3)	C13—C14	1.383 (3)
N4—H41	0.8800	C13—H13	0.9300
N4—H42	0.8800	C14—H14	0.9300
C1—C6	1.393 (3)	C15—C16	1.514 (3)
C1—C2	1.398 (2)	C15—H15A	0.9700
C1—C7	1.458 (3)	C15—H15B	0.9700
C2—C3	1.371 (3)	C16—H16A	0.9700
C2—H2	0.9300	C16—H16B	0.9700
C3—C4	1.385 (3)	O2—C17	1.486 (2)
C3—H3	0.9300	O2—H2O	0.8400
C4—C5	1.368 (3)	C17—H17A	0.9600
C4—H4	0.9300	C17—H17B	0.9600
C5—C6	1.400 (3)	C17—H17C	0.9600
			
C7—N1—C8	121.23 (15)	N2—C8—N1	124.28 (16)
C7—N1—C15	116.50 (15)	N3—C8—N1	114.57 (16)
C8—N1—C15	122.13 (15)	C14—C9—C10	119.07 (18)
C8—N2—C6	117.49 (16)	C14—C9—N3	124.37 (17)
C8—N3—C9	127.54 (17)	C10—C9—N3	116.44 (17)
C8—N3—H31	116.2	C11—C10—C9	120.53 (19)
C9—N3—H31	116.2	C11—C10—H10	119.7
C16—N4—H41	109.5	C9—C10—H10	119.7
C16—N4—H42	109.5	C12—C11—C10	120.61 (19)
H41—N4—H42	109.5	C12—C11—H11	119.7
C6—C1—C2	120.49 (17)	C10—C11—H11	119.7
C6—C1—C7	118.89 (16)	C11—C12—C13	119.0 (2)
C2—C1—C7	120.62 (18)	C11—C12—H12	120.5
C3—C2—C1	120.45 (19)	C13—C12—H12	120.5
C3—C2—H2	119.8	C14—C13—C12	121.1 (2)
C1—C2—H2	119.8	C14—C13—H13	119.5
C2—C3—C4	119.24 (17)	C12—C13—H13	119.5
C2—C3—H3	120.4	C9—C14—C13	119.66 (19)
C4—C3—H3	120.4	C9—C14—H14	120.2
C5—C4—C3	120.99 (19)	C13—C14—H14	120.2
C5—C4—H4	119.5	N1—C15—C16	114.39 (17)
C3—C4—H4	119.5	N1—C15—H15A	108.7
C4—C5—C6	120.9 (2)	C16—C15—H15A	108.7
C4—C5—H5	119.6	N1—C15—H15B	108.7
C6—C5—H5	119.6	C16—C15—H15B	108.7
N2—C6—C1	122.70 (17)	H15A—C15—H15B	107.6
N2—C6—C5	119.27 (17)	N4—C16—C15	111.46 (19)
C1—C6—C5	117.96 (16)	N4—C16—H16A	109.3
O1—C7—N1	120.87 (17)	C15—C16—H16A	109.3
O1—C7—C1	123.97 (17)	N4—C16—H16B	109.3
N1—C7—C1	115.15 (16)	C15—C16—H16B	109.3
N2—C8—N3	121.13 (18)	H16A—C16—H16B	108.0
			
C6—C1—C2—C3	−1.1 (3)	C6—N2—C8—N3	−175.64 (16)
C7—C1—C2—C3	178.97 (17)	C6—N2—C8—N1	2.5 (3)
C1—C2—C3—C4	0.7 (3)	C9—N3—C8—N2	−9.1 (3)
C2—C3—C4—C5	0.1 (3)	C9—N3—C8—N1	172.63 (16)
C3—C4—C5—C6	−0.5 (4)	C7—N1—C8—N2	−6.1 (3)
C8—N2—C6—C1	1.8 (3)	C15—N1—C8—N2	169.49 (18)
C8—N2—C6—C5	178.68 (18)	C7—N1—C8—N3	172.12 (15)
C2—C1—C6—N2	177.50 (16)	C15—N1—C8—N3	−12.3 (2)
C7—C1—C6—N2	−2.5 (3)	C8—N3—C9—C14	−31.8 (3)
C2—C1—C6—C5	0.6 (3)	C8—N3—C9—C10	152.29 (18)
C7—C1—C6—C5	−179.41 (17)	C14—C9—C10—C11	−1.4 (3)
C4—C5—C6—N2	−176.84 (19)	N3—C9—C10—C11	174.75 (17)
C4—C5—C6—C1	0.2 (3)	C9—C10—C11—C12	0.6 (3)
C8—N1—C7—O1	−176.29 (16)	C10—C11—C12—C13	0.3 (3)
C15—N1—C7—O1	7.9 (3)	C11—C12—C13—C14	−0.5 (3)
C8—N1—C7—C1	4.9 (2)	C10—C9—C14—C13	1.2 (3)
C15—N1—C7—C1	−170.91 (15)	N3—C9—C14—C13	−174.6 (2)
C6—C1—C7—O1	−179.64 (17)	C12—C13—C14—C9	−0.3 (3)
C2—C1—C7—O1	0.3 (3)	C7—N1—C15—C16	−96.6 (2)
C6—C1—C7—N1	−0.9 (2)	C8—N1—C15—C16	87.6 (2)
C2—C1—C7—N1	179.07 (16)	N1—C15—C16—N4	−77.5 (2)

### Hydrogen-bond geometry (Å, °)

**Table:**

*D*—H···*A*	*D*—H	H···*A*	*D*···*A*	*D*—H···*A*
N3—H31···N4	0.88	2.04	2.811 (3)	146
N4—H41···O2^i^	0.88	2.13	2.990 (6)	168
O2—H2O···O1	0.84	2.01	2.755 (6)	147

Symmetry codes: (i) *x*, −*y*+1, *z*−1/2.

## References

[bb1] Barbour, L. J. (2001). *J. Supramol. Chem.* **1**, 189–191.

[bb2] Bruker (2007). *APEX2* and *SAINT*. Bruker AXS Inc., Madison, Wisconsin, USA.

[bb3] Sheldrick, G. M. (2008). *Acta Cryst.* A**64**, 112–122. 10.1107/S0108767307043930 18156677

[bb4] Westrip, S. P. (2009). *publCIF*. In preparation.

[bb5] Yang, X.-H., Chen, X.-B. & Zhou, S.-X. (2009). *Acta Cryst.* E**65**, o185–o186. 10.1107/S160053680804049X PMC2968094 21581640

[bb6] Yang, X.-H., Wu, M.-H., Sun, S.-F., Ding, M.-W., Xie, J.-L. & Xia, Q.-H. (2008). *J. Heterocycl. Chem* **45**, 1365–1369.

